# An Event-Related Brain Potential (ERP) Study of Complex Anaphora in Spanish

**DOI:** 10.3389/fpsyg.2021.625314

**Published:** 2021-03-18

**Authors:** Adrián García-Sierra, Juan Silva-Pereyra, Graciela Catalina Alatorre-Cruz, Noelle Wig

**Affiliations:** ^1^Department of Speech, Language and Hearing Sciences, University of Connecticut, Mansfield, CT, United States; ^2^The Connecticut Institute for the Brain and Cognitive Sciences, Storrs, CT, United States; ^3^Proyecto de Neurociencias, Facultad de Estudios Superiores Iztacala, Universidad Nacional Autónoma de México, Mexico City, Mexico; ^4^Department of Pediatrics, University of Arkansas for Medical Sciences, Little Rock, AR, United States

**Keywords:** pronoun resolution, P200, N400, sentence processing, anaphoric relationship, gendered pronouns, Spanish language

## Abstract

This study examines the event- related brain potential (ERP) of 25 Mexican monolingual Spanish-speakers when reading Spanish sentences with single entity anaphora or complex anaphora. Complex anaphora is an expression that refer to propositions, states, facts or events while, a single entity anaphora is an expression that refers back to a concrete object. Here we compare the cognitive cost in processing a single entity anaphora [*ésta*_feminine_; La renuncia (resignation)] from a complex anaphora [*esto*_neuter_; La renuncia fue aceptada (The resignation was accepted)]. *Ésta* elicited a larger positive peak at 200 ms, and *esto* elicited a larger frontal negativity around 400 ms. The positivity resembles the P200 component, and its amplitude is thought to represent an interaction between predictive qualities in sentence processing (i.e., graphical similarity and frequency of occurrence). Unlike parietal negativities (typical N400), frontal negativities are thought to represent the ease by which pronouns are linked with its antecedent, and how easy the information is recovered from short-term memory. Thus, the complex anaphora recruited more cognitive resources than the single entity anaphora. We also included an ungrammatical control sentence [*éste*_masculine_; La renuncia (resignation)] to better understand the unique processes behind complex anaphoric resolution, as opposed to just general difficulty in sentence processing. In this case, event-related potentials (ERPs) elicited by *éste*_masculine_ and *ésta*_*feminine*_ were compared. Again, *ésta* elicited a larger P200. However, different from the experimental condition, a left anterior negativity (LAN) effect was observed for *éste*; the ungrammatical condition. Altogether, the present research provides electrophysiological evidence indicating that demonstrative pronouns with different morphosyntactic features (i.e., gender) and discourse parameters (i.e., single entity or complex referent) interact during the first stage of anaphoric processing of anaphora. This stage initiated as early as 200 milliseconds after the pronoun onset and probably ends around 400 ms.

## Introduction

### Anaphoric Relation

A pronoun, on its own, does not provide sufficient information to identify the intended referent. Yet, the preceding information (context) can be used to interpret pronouns without great difficulties. This referential relationship is known as anaphora, and allows pronouns to refer back to previously mentioned antecedents (e.g., people, things, events, etc.) without incorporating them into the present context. Further, anaphora can be classified according to the type of antecedent the pronoun refers to. Simply, single entity anaphora is when a pronoun refers back to a concrete object (i.e., people, things, etc.). In contrast, complex anaphora is when a pronoun refers back to abstract concepts (i.e., events, etc., Consten et al., 2007). These relationships exist in many languages, but remains understudied in Spanish. Therefore, the goal of the present study is to investigate the cognitive costs relative to referential resolution of single entity and complex anaphora in the Spanish language.

### Linking Pronoun to Antecedent in Spanish

Spanish, and other languages, can use both personal pronouns and demonstrative pronouns to refer back to the previous context. While personal pronouns (i.e., él/he, ella/she, etc.) are more often used to refer to older topics, demonstrative pronouns (i.e., ésta/this or that, esto/this or that, etc.) are more often used to refer to newly introduced information (Comrie, 1997). Further, it has been shown that personal pronouns slightly prefer grammatical subject antecedents, whereas demonstrative pronouns strongly favor non-subject antecedents in German (Bosch et al., 2007) and in Spanish (González-Álvarez et al., 1997). Sentences 1 and 2 show these phenomena for personal and demonstrative pronouns, respectively.


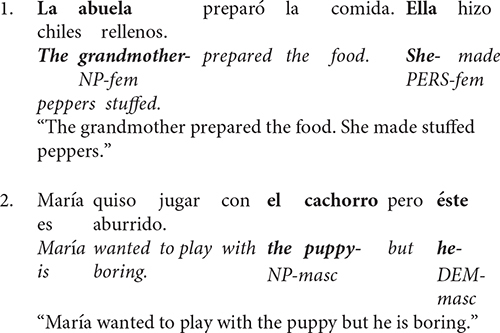


Further, syntactic agreement features of the preceding sentence can be used to identify a pronoun’s correct antecedent. For example, Spanish can use grammatical gender (i.e., male or female) to match a given pronoun with its appropriate antecedent. Sentence 3 shows a situation in which the gender of the pronoun (i.e., female- ésta) constrains the choice of antecedent (i.e., female- la chaqueta vs. male- el saco). In contrast, gender cannot constrain antecedent selection in Sentence 4 since all antecedents share the same gender with the pronoun (i.e., female- mamá vs. female- perrita). However, one antecedent is a person (i.e., mamá) while the other is not (i.e., perrita). Thus, the preference to avoid linking demonstrative pronouns to persons is a more reliable constraint than gender in Sentence 4.


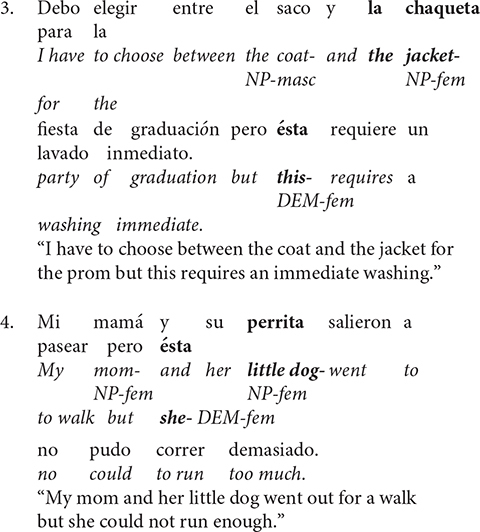


A group of three Spanish demonstrative pronouns (*esto*, *eso* and *aquello*) have been labeled “neuter demonstratives” based on their referential properties (i.e., neither male nor female). Therefore, they are most commonly used to refer to objects that denote abstract concepts like events, facts, situations, etc (Real Academia Española, 2009). This relationship can be observed in sentence 5 below. Specifically, if the speaker intended to refer to the female noun, *La casa* (i.e., The house), they would have used the female demonstrative pronoun *ésta*. But instead, the neuter demonstrative *esto* is used to refer back to the previously mentioned overall event (*The house was locked.*/La casa estuvo cerrada…).


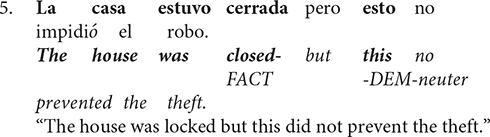


### Complex Anaphora

According to Consten et al. (2007), complex anaphora are nominal expressions that refer to propositions, states, facts or events (propositionally structured referents) introduced as unified entities in a discourse (see sentence 5). The antecedent has to be a complex linguistic entity (consist of at least one clause); and the referent has to be a conceptually complex item (i.e., second order entities- events, processes, states-of- affairs located in time; third order entities- propositions located outside time and space; Lyons, 1977: 443). This type of anaphoric relationship is also called abstract object anaphora (Asher, 1993, 2000), reference to fact (Halliday and Hasan, 1976), or discourse deixis (Webber, 1991).

Spanish uses both gendered and neuter demonstrative pronouns (“this/that”), but in different ways. Gendered demonstrative pronouns, like *éste* or *ésta*, often refer to inanimate objects with an assigned gender (single entity anaphora). In contrast, neuter demonstrative pronouns, like *esto*, often refer to less defined entities like events, facts or situations introduced via clauses or sequences of clauses. Simply, neuter demonstratives in Spanish imply a complex anaphoric relationship between antecedent and the pronoun (Consten et al., 2007). Thus, gendered and neuter demonstrative pronouns in Spanish imply different anaphoric relationships.

The differences between single entity anaphora and complex anaphora in Spanish can be seen across Sentence 6. Explicitly, Sentences 6a and 6b provide an appropriate discourse referent (i.e., la avioneta and ésta, respectively) for an object antecedent, *avioneta* (single entity anaphora). Yet, Sentence 6c does not. Instead, the neuter demonstrative pronoun *esto* infers a discourse referent that is an event (i.e., hijack of the plane; complex anaphora).


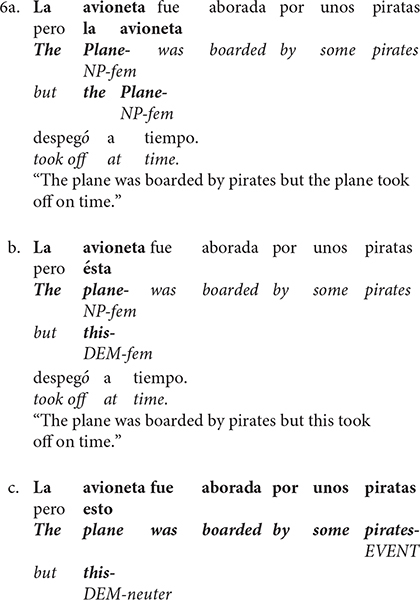



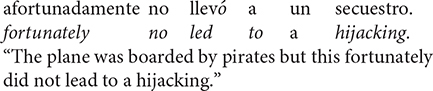


Consten et al. (2007) claim that references to complex objects imply relationships between different ontological types in a linear hierarchy. Specifically, they suggest that the degree of abstractness increases along the following line: event < process < state < fact < proposition. Similarly, Givón (1984) proposes that states and events are experiences of variable stability, such that defining entities are temporally more stable than events. Also, entities are thought to be less abstract than events since they can be physically encoded, referring to spatially delimited entities, while events only exist in time (Givón, 1979).

Finally, Consten et al. (2007) describe the change in the level of abstraction from a previously mentioned referent, to a new type of discourse object, as the anaphoric complexation process. Anaphoric complexation can shift referents from one abstraction level to a discursive entity of the same, or higher level. However, referents cannot be translated into a discourse entity that is less abstract.

### Approaches to Referential Resolution

It is generally agreed that there is a correlation between the type of referential form and the level of saliency. Namely, the more accessible a referent is, the less lexical material is needed to form a referential expression. As a consequence, pronouns that become associated with a highly accessible referent create a reduced reference form. Thus, such a reduced anaphoric expression (e.g., unstressed pronoun) requires a prominent referent to be in the reader’s mental model of the discourse and vice versa. This view is considered in the referential form hierarchy models (Givón, 1983; Ariel, 1990, 2001; Gundel et al., 1993). Building on this idea, salience hierarchy-based approaches specifically claim that personal pronouns have more salient antecedents than demonstratives, and that the referential properties of different forms are from their positions on the hierarchy as opposed to differences in informativeness.

Reference resolution, assumed to be an indicator of a referent’s salience, is influenced by word order, thematic role, information structure, anaphoric form, and verb semantics among others (Arnold, 2001; Järvikivi et al., 2005; Kaiser and Trueswell, 2008; Kehler et al., 2008; Schumacher and Hung, 2012). Although, it has been originally assumed that a single factor (as word order, thematic role, etc.) determines salience, nowadays it is more accepted that resolution cannot be reduced straightforwardly to the salience level of the antecedent (Kaiser and Trueswell, 2008). Other points of view regard salience as a compound notion resulting from the interaction between multiple properties of the expression (Kaiser and Trueswell, 2008; Kaiser, 2010, 2011).

An alternative approach that can model the relationship between pronoun interpretation and production is Bayes’ theorem to referential resolution (Kehler et al., 2008; Kehler and Rohde, 2019). Here, interpretative preferences not only depend on the prominence structure of previous discourse, but also arise from the combination of comprehenders’ expectations and estimations. Thus, comprehenders use the prior discourse to form predictions about which referent is most likely to be mentioned again in the discourse. Once an anaphora is found, they update their prediction by integrating their initial predictions with the referential bias (evidence) provided by the form of the anaphora.

### Event-Related Potentials

Event-related potentials (ERPs) can assess cognitive processes that occur in the range of milliseconds, and thus are a powerful tool for analyzing the chronology of discourse integration as in anaphoric processing (see for review Callahan, 2008). Some findings suggest that an attempt to locate the correct antecedent can occur as early as 280 ms after a pronoun’s presentation (van Berkum et al., 1999a). Specifically, early negativities in the ERP response are seen when antecedents are selected using morpho-syntactic constraints (e.g., gender, number, case agreement; Demestre et al., 1999; Lamers et al., 2006, 2008). Yet, if the agreement features of the anaphor are incompatible with the only possible antecedent (i.e., syntactic violation), the anaphor elicits a brain response known as the Left Anterior Negativity (LAN) 300–500 ms after the onset of the grammatical violation (Molinaro et al., 2011).

However, when there are two possible referents and thus more difficult to select the correct antecedent, a sustained anterior negativity is observed post-onset of the anaphor (van Berkum et al., 1999a,b, 2003b; Dwivedi et al., 2006; Nieuwland and van Berkum, 2006; Nieuwland et al., 2007), which is distinct from the N400 ERP component discussed below (van Berkum et al., 1999a). The anterior negativity may be defined as a referential negativity, a component that marks the memory retrieval of the antecedent. This effect could be observed in Sentence 4 since the identification of the antecedent depends on pragmatic information (i.e., both possible antecedents are female, but one antecedent is more appropriate for the following context).

A negativity with a parietal distribution between 500 and 600 ms post word onset (i.e., N400) also has been observed when there is a difficulty in establishing an anaphoric relation (Streb et al., 1999, 2004; Burkhardt, 2005, 2006). Previous research has shown that the N400 amplitude increases as a function of contextual expectation (Kutas and Federmeier, 2011). Explicitly, the less expected a critical word is within a given context (e.g., word list, sentence, and discourse context), the larger the N400 amplitude becomes. Thus, the N400 is thought to reflect the difficulty in integrating a word into a semantic or discourse representation, and serves as an indicator for semantic processing (Kutas et al., 2000; Kutas and Federmeier, 2011). In accordance with this interpretation, the N400 also has been associated with the processing of the anaphoric relationship. It has been reported that increasing distance between the anaphor and its antecedent demands a longer processing time (Streb et al., 1999, 2004), aptly represented by a larger N400 during pronominal resolution. This respective modulation of N400 could reflect the difficulty of integrating an anaphoric expression into a representation of mental speech (Streb et al., 1999, 2004). Simply, the further away the antecedent, the harder it is to detect, and then integrate (i.e., larger N400).

Also, a larger N400 has been observed in the absence of an identity relationship between the anaphora and its referent. Burkhardt (2006) investigated the processing of anaphora (i.e., givenness) in German by comparing sentences with different types of relation to the referent: direct anaphora (i.e., identity relationship; 7a), indirect anaphora (7b), and discourse-new expressions (7c).


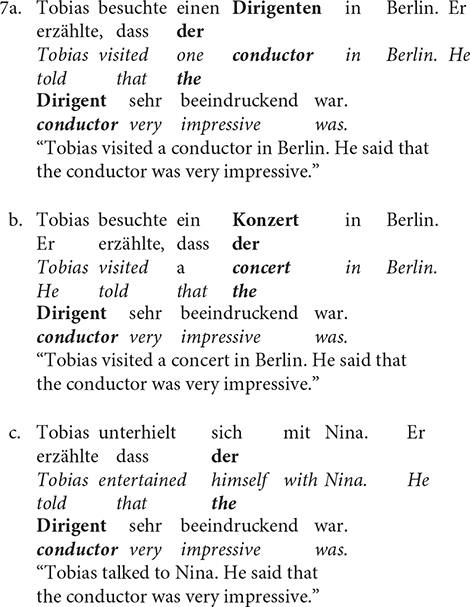


The types of anaphoric resolution seen in Sentence 7b (the relationship is established when the referent information is inferred) and 7c (new information is introduced and there is no referent) elicited a greater amplitude of the N400 than that in 7a (where the referent and the anaphor are the same). Even more, Sentences 7b and 7c, compared to 7a, elicited a larger amplitude of a late positivity (i.e., P600) due to the higher demands arising from the establishment of an independent discourse referent and successive storage demands.

The late positivity (or P600), which has been originally observed when using paradigms of syntactic violations (Friederici, 1997), is elicited over the centro-parietal scalp regions around 400 to 1000 ms post word-onset. However, since the late positivity also can be elicited in the absence of syntactic violations, it has been hypothesized to depict the difficulty in integration or interpretative brain processes (for a review see Sassenhagen et al., 2014). For the purpose of the present study, we take the perspective of referential processing such that the late positivity will reflect an update in the mental model. Specifically, the addition of new information or discourse units (or the modification of previously established structures) demands an update in the mental model; thus, increasing the difficulty of the integration process (Kaan et al., 2000, 2007; Burkhardt, 2006, 2007; Kaan, 2007; Hung and Schumacher, 2012). This also agrees with the idea that the late positivity reflects processes associated with the maintenance and updating of discourse representation structure (Coulson et al., 1998; Kaan et al., 2007; Bornkessel-Schlesewsky et al., 2011), which applies to pronoun resolution.

### ERP Studies of Complex Anaphora

Marx et al. (2007) investigated the cognitive processing of complex anaphoras. They wanted to know if there was a greater cognitive effort in the processing of complex anaphoras than anaphoras that have noun phrases (NP) as their antecedent. They used sentences of the type:


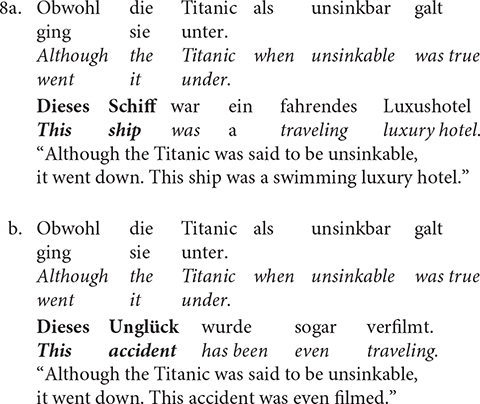


Marx et al. (2007) found that the complexity of the anaphora did not modulate N400 amplitude. This indicated that the complexation process required for the integration of ***This accident*** in Sentence 8b did not exert any additional cognitive cost. Specifically, the cognitive effort behind the anaphoric process of linking an expression in a previously introduced entity does not differ between referring to a specific object as in 8a, or a propositionally structured entity as in 8b. However, when Marx et al. (2007) separated the NP-anaphora as a function of the syntactic structure of the context, they observed a different pattern. Marx et al. (2007) separated the experimental material (stimuli) into two groups depending on whether the NP-anaphorical expression referred to the subject, or the object of the preceding sentence (i.e., syntactic role). The new analysis showed that complex anaphors, elicited a larger late positivity than NP-anaphors. Thus, it can be deduced from this study that the late positivity can be interpreted as an indicator for cognitive effort while introducing a new discourse entity in the mental representation. Similarly, other studies have shown that the late positivity is modulated by sentential position or topicality (Schumacher and Hung, 2012).

Schumacher et al. (2010) carried out an ERP study to explore how processing strategies changed as the complex anaphoric reference varied in degree of abstractness (ontological configuration). For this purpose, sentences as 9a (no change of abstractness between antecedent and anaphora), 9b (increasing abstractness from antecedent to anaphora) and 9c (decreasing abstractness from antecedent to anaphora; a violation of the abstractness constraint) were compared.


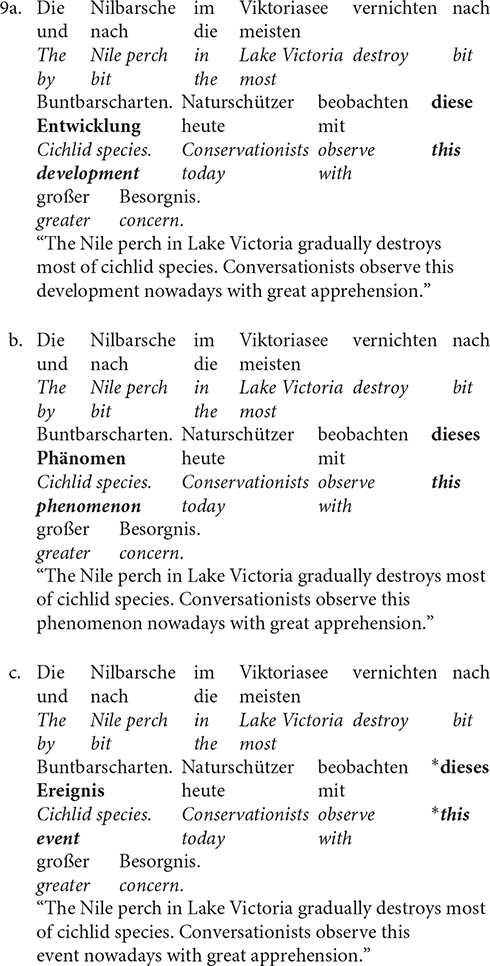


In this study, an N400 was found, but not in all conditions. The results revealed that increasing abstractness (9b) did not lead to a greater negativity when compared to no change of abstractness (9a). Instead, an unexpected larger P200 to sentences with increasing abstractness was observed (i.e., 9b > 9a). Further, sentences with decreasing abstractness (9c) elicited an enhanced centro-parietal negativity, or the N400. In other words, a violation of the abstractness constraint creates higher costs in processing demands when integrating information.

The P200 has previously been shown to be sensitive to physical features (e.g., Luck and Hillyard, 1994; Barber et al., 2004; Dambacher et al., 2006; Evans and Federmeier, 2007), as well as other factors beyond perceptual processing, such as if the action of the verb is being completed or not (i.e., telicity see: Malaia et al., 2009). In Schumacher et al. (2010), a P200 was reported even after controlling for word length and word frequency. Schumacher and colleagues propose that the P200 was associated with a higher degree of abstractness. However, more recent discourse-related studies have found an enhanced P200 for sentences that induced a new word relative to those that continued the topic (i.e., givenness; Hung and Schumacher, 2012, Hung and Schumacher, 2014). In this regard, a proposed interpretation of the P200 is that it is sensitive to word repetition. Specifically, a smaller P200 is associated with repeated words in the topic condition (Burmester et al., 2014), and with processing similar graphical forms as an early perceptual mismatch response.

In general, the electrophysiological data suggest that the N400 and late positivity ERP components are modulated by referential expressions that differ in their degree of givenness (given or new information), abstractness (level of abstractness) and syntactic role (i.e., reference to the subject or to the object). For instance, both givenness and syntactic role are powerful cues to the saliency of a referential expression. Thus, these aspects impact the expectations created by the context during referential processing, and modulate the N400. In contrast, referential expressions with more complex structure seem to modulate the late positivity. In this sense, since topicality can structure discourse representation (by signaling what an utterance is about, and hence identifying the discourse unit relative to which information is to be stored), entities at non-initial positions have an impact on the late positivity.

The ERP data support the syntax-discourse model (SDM) (Avrutin, 1999; Burkhardt, 2005, 2006; Schumacher, 2009). This model considers two operations (Discourse-Linking and -Updating) for the construction of the discourse representation. First, Discourse-Linking operates to link an incoming referential expression with prior discourse. This operation is a function of the antecedent features (syntactic and grammatical function, morphosyntactic form, etc.) and of the discursive-pragmatic parameters. This means that the salience of a referential expression influences the linking operation. For instance, the salience computation is impacted by givenness and topicality. Second, discourse representation structure is assessed, and if necessary, updated (Discourse-Updating). When new discourse units must be established in the discourse representation, or when previously built structures must be reanalyzed or enriched (for instance as a result of inferencing), discourse-internal operations are required.

### The Present Study

The goal of the present study is to investigate if the cognitive cost of referential resolution for complex anaphoras is higher relative to the referential resolution of anaphora that refers to an entity in Spanish. By using context-clause and demonstrative pronoun-clause sentences (as 10; see below), we compared the ERPs to those sentences that include a gendered pronoun that refers to NP-antecedent (10a) with those that include a neuter demonstrative pronoun (10b) that refers to an event represented in the first clause. Further, we included a disagreement between a gendered pronoun and antecedent as in sentence 10c to create an ungrammatical sentence.


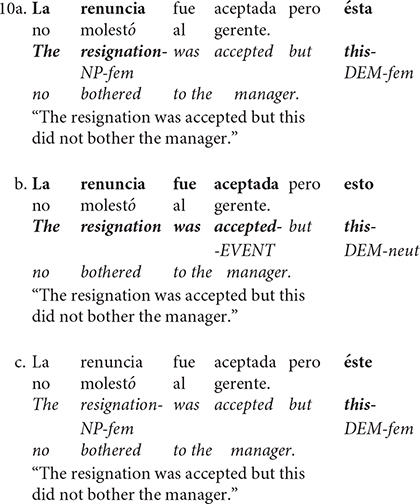


Since variations in the position of the topic in the sentence have an important effect on the discourse updating, we created our sentences to have exactly the same structure. Explicitly, our stimuli consisted of two simple sentences joined with a coordinated conjunction (i.e., but), with different Spanish demonstrative pronouns (i.e., ésta, esto, éste) in the same position across a set of sentences (10a vs. 10b vs. 10c). Since the demonstrative pronoun has a preference for the object and can even exclude the subject for the referential resolution, we opted for a non-canonical structure in Spanish described below.

The context is a simple, predicative, passive, impersonal, enunciative, affirmative sentence (10a). There is an unpronounced agent, and a patient who is the subject of the sentence. The target sentence (10b) has the neuter demonstrative pronoun and is also a simple, predicative and enunciative but is active, transitive, and negative sentence. Intuitively, we would expect that referential resolution of complex anaphora (sentences with neuter demonstrative as 10b) requires a greater cognitive effort than for anaphora that refers to an entity (sentences with gendered demonstrative as 10a). This is due to the fact that complex anaphors require a propositionally structured object to be established as a referent, whereas gendered demonstrative anaphors (10a) already have an existing referent that is reactivated by the gender. The degree of abstractness should represent a powerful cue for Discourse Linking with prior discourse (reflected in N400-modulations: 10b > 10a) because the salience of these referential expressions influences the expectations generated by the context during referential processing (such as givenness: inferred vs. given). However, based on previous findings from referential processing of complex anaphora in which no N400 modulations were observed, but have demonstrated effects on discourse updating with respect to the computation of prominence features (Marx et al., 2007 extended analysis), we could expect an amplitude modulation of the late positivity (10b > 10a). Such a pattern would suggest that no extra resources were required in the linking operation, but were for discourse-updating.

Ungrammaticality is modeled (10c) as a gender disagreement between the pronoun and its only possible antecedent (i.e., male pronoun and female antecedent). In terms of morphosyntactic features, this experimental condition would be similar to the gender disagreement of the neuter demonstrative relative to its antecedent (10b). In this scenario, a LAN followed by a late positivity is expected for the ungrammatical stimuli (10c) compared to the grammatical sentences (10a; Molinaro et al., 2011). For the purpose of the present research, we used it as a control scenario.

## Materials and Methods

### Participants

Twenty-five (15 female) Spanish speaking young adults were recruited from the psychology school at the National University of Mexico. All participants were healthy (with no history of neurological or psychiatric disorders) and around 19 years old (mean age = 19.62 years old; *SD* = 0.97; range = 18 to 22 years old). All subjects included in the analyses were right-handed as assessed by an abridged Spanish version of the Edinburgh Handedness Inventory (Oldfield, 1971): LQ > + 50. All subjects had no family history of left-handedness. All participants were informed of their rights and provided written informed consent for participation in the study. This research was carried out ethically and was approved by the Ethics Committee of Universidad Nacional Autónoma de México (Ethical Application Ref: CE/FESI/062020/1299).

### Stimuli

Nouns and verbs from ESPAL^1^ were used to build 720 experimental sentences using three anaphoric demonstrative pronouns: ésta/esto/éste (i.e., 240 sentences for each). All sentences were between 9 and 10 words in length. Additionally, 1,080 noun–verb number agreement sentences (540 agree and 540 disagree) sentences were included as filler sentences. Filler sentences are employed as experimental sentences elsewhere. A Latin square was used to create six lists of materials and to ensure that each sentence occurred in each of the within-materials conditions.

Each list contained 300 sentences: 120 experimental (40 grammatical entity anaphora sentences with *ésta*; 40 grammatical complex anaphora sentences with *esto*; 40 ungrammatical entity anaphora sentences with *éste*: see below examples 10 a-c) and 180 filler sentences (70 grammatical and 110 ungrammatical sentences). From the 300 sentences, half of the sentences were correct Spanish sentences, and the other half were ungrammatical sentences (150 grammatical and 150 ungrammatical sentences). Finally, the six lists were counterbalanced (see Supplementary Appendix A for an example of a list).

### Procedure

Stimuli were delivered by Stim2 software (CompuMedics NeuroScan, Charlotte, NC, United States). A fixation point (“+”) appeared in the center of the screen and remained there for 2700 ms. This fixation point was followed by a blank screen interval of 300 ms. Then, the sentence was displayed word by word, where each word appeared for 300 ms and was followed by a 300 ms blank interval.

Participants were required to do a grammatical judgment at the end of each sentence. A question mark appeared at the end of each sentence to indicate participants to give their response. The question mark remained for 2,000 ms or until the participant responded. They could press the left mouse button to indicate that the sentence was grammatically correct or the right button to indicate that the sentence was incorrect. Response buttons were counterbalanced among subjects. The inter-trial interval between the end of the grammatical judgment and the presentation of a new sentence varied randomly between 1,000 and 1,500 ms.

### ERP Recording and Analysis

The EEG was recorded from 64 tin electrodes embedded in a standard quick-cap, each referenced on-line to the left mastoid. Data were re-referenced off-line by the average signal of left and right mastoids. Blinks and eye movements were monitored through a bipolar recording from two electrodes placed on the outer canthi of each eye and four above and under each eye. Electrode impedances were maintained below 10 kOhms. The EEG was amplified with the NeuroScan SynAmps system and Scan 4.5 software (CompuMedics, NeuroScan) with band pass set from 0.1 to 100 Hz and sampled at a rate of 250 Hz. Trials with artifacts due to eye movements, excessive muscle activity, or amplifier blocking were eliminated off-line before averaging—approximately 5% of the data for each target pronoun (with roughly equal loss of data across conditions).

Event-related potentials were time-locked to the onset of the pronoun and were computed off-line from 1,200 ms epochs for each subject in each experimental condition. Epochs consisted of the 200 ms preceding, and 1000 ms following the presentation of the individual critical word in each sentence. Automatic rejection of segments was carried out based on the following criteria: segments with electrical activity exceeding ± 100 mV, and amplifier blocking for more than 50 ms at any electrode site were considered artifacts and the entire segment was rejected. The ocular artifact reduction tool provided by the Scan 4.5 software was used. Subjects with fewer than 30 artifact-free trials for each condition were excluded from the average. Baseline correction was performed using the 200 ms pre-stimulus time window. There were no differences in the number of segments between experimental conditions (i.e., *ésta, esto, éste*).

### Data Analysis

Percentages of correct responses and means of reaction times (RT) from correct responses of task performance (grammatical judgment) were included in the behavioral analyses. Paired *t*-tests with non-parametric permutation analyses were performed using these behavioral data to compare *esto versus ésta* and *éste versus ésta.*

Event-related potential amplitude analyses were done with BESA Statistics 2.0 (BESA GmbH, Gräfelfing, Germany), which uses data clustering in combination with permutation testing. This process is a data-driven approach that assumes if a statistical effect is observed in an extended period of time in several neighboring channels, then it is unlikely that the effect occurred by chance. In our experiment we examined the time window from 0 to 700 ms after stimulus onset in 64 electrodes (11,200 data points). In the first step, BESA performs a parametric test to find data clusters that show pronounced effects. BESA calculates a cluster-value for each pronounced effect that represents the sum of the *t*-values in the time (ms) and spatial domain (electrodes) in which *p*-values are below 0.05. Therefore, a large cluster-value represents a significant difference in the time domain across multiple neighboring electrodes, while a small cluster-value represents a significant difference in one or few electrodes. In the present research, we used a channel neighbor distance of 4.5 cm.

In the second step, BESA repeats step 1, but using a permutation test. This serves to test if the probabilities of the cluster-values across experimental conditions (or subjects) are exchangeable. Hence, for each of the calculated permutations (in our case 10,000), a new *t*-test is computed per data-point, and new clusters are determined. Accordingly, each permutation will result in new cluster-values for each cluster. Thus, a distribution of cluster-values can be established across all permutations and the α-error of the initial cluster-value in step 1 can be directly determined. In other words, it is determined if the initial cluster-value derived in step 1 is equally likely to occur as any other cluster-values derived in each permutation step. These types of analyses are performed to control for Type I error due to the large number of data points compared in ERP responses (see: Bullmore et al., 1999; Ernst, 2004; Maris and Oostenveld, 2007).

The ERP comparisons of interest are *esto* vs. *ésta* and *éste* vs. *ésta*. The last comparison would reveal the ERP components associated with morphosyntactic and repair analysis when the sentence contains a syntactic violation.

## Results

### Behavioral Data

Table 1 shows behavioral means and standard deviations. There was no evidence of differences in responses times between *esto* and *ésta* (*t* = −0.67, *p* = 0.24), but the correct responses to *esto* were significantly longer than those to *ésta* (*t* = 2.94, *p* = 0.004). Regarding the comparison of the control condition (*éste*) with *ésta*, the responses to *ésta* were significantly longer (*t* = 3.94, *p* = 0.0006), but no differences were found in the percent of correct responses between these pronouns (*t* = 0.17, *p* = 0.42).

**TABLE 1:** Means and standard deviations of reaction times and percentage of hits.

Demonstrative pronoun	Mean (*SD*)	
	
	RT	%
esto … [this *_*neuter*_*]	791.2 (209.2)	81.9 (10.7)
ésta … [this *_*feminine*_*]	804.9 (194.9)	75.5 (10.1)
éste … [this *_*masculine*_*]	705.4 (184.6)	74.8 (15.6)

### ERP Data

The comparisons were done using BESA Statistics 2.0 (BESA GmbH, Gräfelfing, Germany) in the time interval between 0 to 700 ms in 64 scalp electrodes.

#### *esto* vs. *ésta*

**FIGURE 1:**
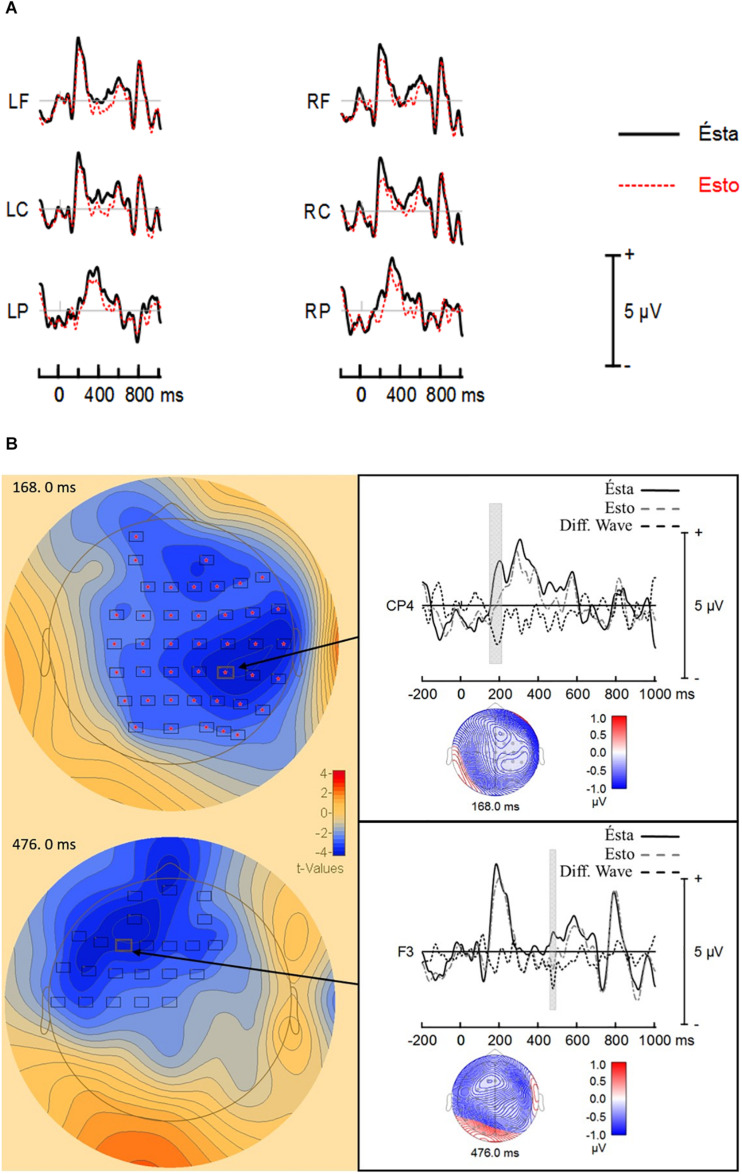
**(A)** ERP grand averages. Electrode regions are Left Frontal (LF = FP1, AF3, F7, F5, F3, and F1), Left Central (LC = FC5, FC3, FC1, C5, C3, and C1), Left Parietal (LP = CP5, CP3, CP1, P5, P3, and P1), Right Frontal (RF = FP2, AF4, F8, F6, F4, and F2), Right Central (RC = FC5, FC4, FC2, C6, C4, and C2), and Right Parietal (CP6, CP4, CP2, P6, P4, and P2). **(B)** Significant electrode cluster obtained by BESA statistics in the first analysis. The left side of the figure shows *t*-values map at the maximum amplitude difference between *ésto* and *ésta*. The right side of the figure shows the ERPs responses and difference waveforms (*ésto* minus *ésta*). The gray bar in the ERP responses represents the time window in which both responses differed in a significant way for CP4 and F3. Voltage maps are time locked to the most significant amplitude difference in the difference waveform. pronoun resolution, P200, N400.

The analysis showed a summation of individual *t*-values (cluster-value) of −1038.76 from 148 to 220 ms after stimulus onset. The cluster-value showed a different probability distribution between *esto* and *ésta* (*p* = 0.008). Hence, the results demonstrated a significant difference between *ésto* (Mean = 0.201 μV; *SD* = 1.24) and ésta (Mean = 1.11 μV; *SD* = 1.34). Figure 1A shows a less positive ERP response to *esto* than to *ésta* in central and right frontal-central electrodes, which indicates *ésta* elicited a larger P200 than *esto*.

The analysis showed a second cluster-value of −461.26 from 432 to 496 ms after stimulus onset. The cluster-value showed a significant trend in the probability distribution between *esto* and *ésta* (*p* = 0.06). This means that there was a significant trend between *esto* (Mean = −0.186 μV; *SD* = 1.06) and *ésta* (Mean = 0.700 μV; *SD* = 0.940). Figure 1A shows a less positive ERP response to *esto* than to *ésta* in left frontal-central electrodes, which suggests *esto* elicited a larger frontal negativity (i.e., distinct from the LAN and N400) than *ésta*. Figure 1B shows the ERP grand averages by electrode regions.

#### *éste* vs. *ésta*

**FIGURE 2:**
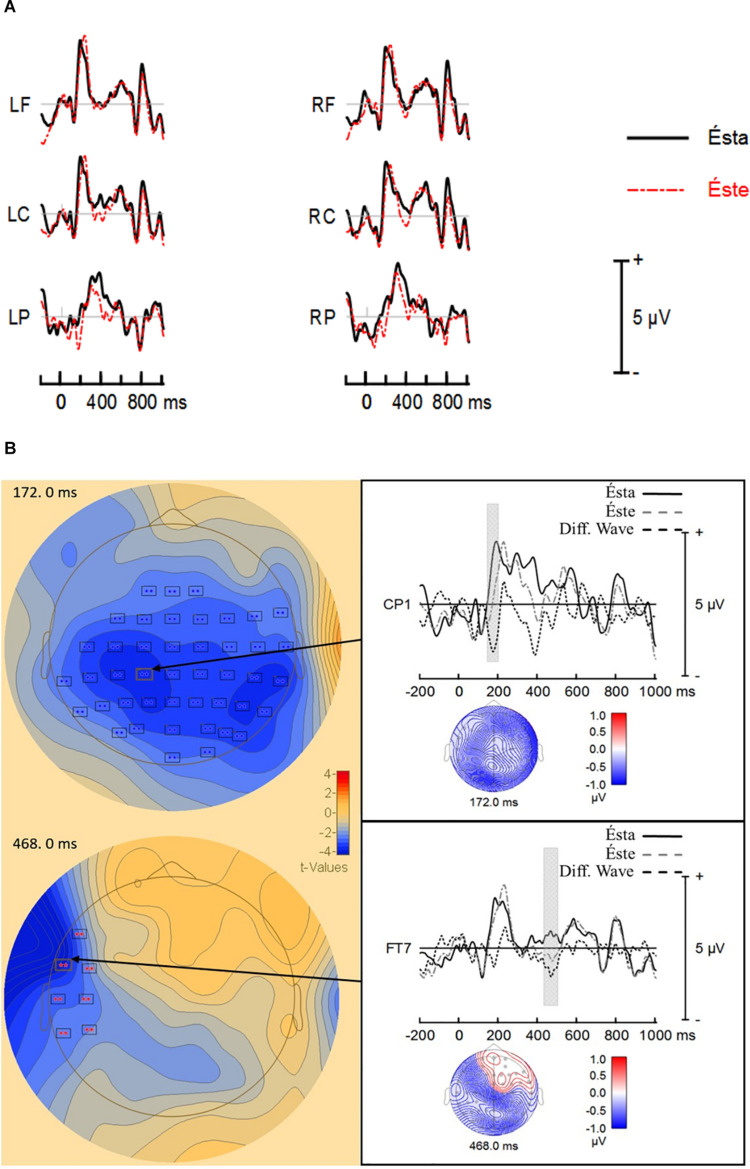
**(A)** ERP grand averages. Electrode regions are Left Frontal (LF = FP1, AF3, F7, F5, F3, and F1), Left Central (LC = FC5, FC3, FC1, C5, C3, and C1), Left Parietal (LP = CP5, CP3, CP1, P5, P3, and P1), Right Frontal (RF = FP2, AF4, F8, F6, F4, and F2), Right Central (RC = FC5, FC4, FC2, C6, C4, and C2), and Right Parietal (CP6, CP4, CP2, P6, P4, and P2). **(B)** Significant electrode clusters obtained by BESA statistics. The left side of the figure shows *t*-values maps at the maximum amplitude difference between *éste* and *ésta*. The right side of the figure shows the ERPs responses and difference waveforms (*éste* minus *ésta*). The gray bar in the ERP responses represents the time window in which both responses differed in a significant way for CP1 and FT7. Voltage maps are time locked to the most significant amplitude difference in the difference waveform. pronoun resolution, P200, N400.

The analysis showed a summation of individual *t*-values (cluster-value) of −1225.13 from 148 to 256 ms after stimulus onset. The cluster-value showed a different probability distribution between *éste* and *ésta* (*p* = 0.0016). Hence, the results demonstrated a significant difference between *éste* (Mean = −0.570 μV; *SD* = 1.24) and *ésta* (Mean = 0.533 μV; *SD* = 1.18). Figure 2A shows a less positive ERP response for *éste* than to *ésta* in left- and right-central-parietal electrodes, which indicates *ésta* elicited a larger P200 than *éste*.

The analysis showed a second cluster-value of −1638.35 between 308 and 504 ms. The cluster-value showed a different probability distribution between *éste* and *ésta* (*p* = 0.0005). Hence, the results demonstrated a significant difference between *éste* (Mean = 0.170 μV; *SD* = 1.12) and *ésta* (Mean = 1.16 μV; *SD* = 1.22). Figure 2A shows a less positive response for éste in left-frontal electrodes, which indicates éste elicited a larger LAN than *ésta*. Figure 2B shows the ERP grand averages by electrode regions.

## Discussion

The present study was designed to assess reference resolution of complex anaphora. Here, complex anaphora in our target active sentences was created by locating the pronouns in the subject position (i.e., previous context passive sentence with subject-patient). Sentences included either neuter or gendered demonstrative pronouns in Spanish. Specifically, we compared ERPs to the neuter demonstrative pronoun *esto* (that is used to refer back to an event) with the gendered demonstrative pronoun *ésta* (that is used to refer to only one entity). We expected that integrating a complete clause (*esto* referring to an event) into the discourse would generate a higher cost in the processing of the referential resolution than integrating an entity (*ésta*); thus, modulating the late positivity (discourse updating operation). However, our results did not show the amplitude modulation for the late positivity. In contrast, our results showed a frontal negativity peaking within the N400 time-range, but distinct from both the N400 and LAN. Additionally, our results showed a P200 amplitude modulation for the processing of the demonstrative pronouns. Our results and implications are discussed below.

### The Late Positivity

The late positivity is observed in centro-parietal electrodes around 600 ms after stimulus onset, and aptly has been observed in syntactic violation paradigms (Friederici, 1997). However, beyond syntactic violation resolution, research suggests that the late positivity depicts the difficulty in integrating information or interpretative brain processes (see Sassenhagen et al., 2014). Thus, modulation of the late positivity is considered an index of anaphoric integration cost due to the establishment of an independent, new discourse referent (Burkhardt, 2006, 2007). In the present investigation, we expected modulations in the late positivity as a function of difficulty in discourse integration. Namely, we expected a larger late positivity for the more difficult integration of *esto* (complex anaphora) compared to that of *ésta* (entity anaphora).

However, we did not find this expected amplitude modulation for the late positivity. We propose that the absence of this effect was due to how our sentences did not create a topic change that would require a discourse update to resolve the reference. Specifically, all our target sentences included a demonstrative pronoun as anaphora, whether gendered or neuter. The gendered demonstrative’s antecedent was a single entity, while the neuter demonstrative’s antecedent was a complex object (a clause). In the case of the gendered demonstrative (*ésta*), was expected to trigger the linking operation between the demonstrative and the antecedent (N400), as well as a discourse update (late positivity). The same process was expected for the neuter demonstrative (*esto*), but unlike the gendered demonstrative, *esto* was expected to link the entire preceding context (discourse-updating) and hence a larger cost in the discourse updating was expected when compared with *ésta.* However, we did not find a larger late positivity for *esto* than *ésta*. Our results are in accordance with Marx et al. (2007) who did not find a modulation of the late positivity when using complex anaphora. However, in a deeper exploration of their data, they showed that complex anaphora elicits a larger late positivity when compared with NP-anaphoric expressions that refer to subjects, but no amplitude differences were observed when compared with NP-anaphoric expressions that refer to objects. Marx et al. (2007) research suggests that we did not observe a difference in the positive amplitude due to the syntactic role. Namely, in our case, the absence of an amplitude difference in the late positivity could point to presence of the effect in both conditions. This fact would generate a similar cost, although due to different reasons. That is, ésta might elicit a late positivity due to being a demonstrative pronoun, which signaling a referential shift (the covert agent argument was the previous topic) and esto additionally might elicit a late positivity due to complexation. This interpretation would support the idea that referential processing is modulated by the topic principle sentence-initially, whereas non-initial positions are operated under the given-new consideration (Hung and Schumacher, 2012; Schumacher and Hung, 2012). As pointed by one of the reviewers in the present article, another explanation for not observing a late positive effect is because the demonstrative pronoun was probed, while previous studies involving complexation processes looked at full noun phrases. Therefore, there might be a difference due to the type of referential expression used across experiments.

### Frontal Negativity

Unlike previous referential resolution studies of complex anaphora (Schumacher et al., 2010), our results for *esto* vs. *ésta* showed no parietal N400 response. Instead, *esto* produced a left fronto-central negativity around 400 ms that is similar to the referential negativity reported in ambiguous anaphoric expressions (van Berkum et al., 1999a,b, 2003a,b; Camblin et al., 2007; Nieuwland et al., 2007; Almor et al., 2017). Specifically, a frontal negativity has been observed in situations where referent identification is difficult, either due to the difficulty in matching the anaphor with the correct antecedent to create a new referent, or due to the presence of multiple antecedents that are both equally plausible and simultaneously active in working memory (Camblin et al., 2007; Nieuwland, 2014). This would suggest that the linking operation between the neuter demonstrative (*esto*) and its complex antecedent required an additional operation besides linking the anaphor with its referent.

In contrast, a left anterior negativity (LAN) appears when a sentence is detected to be ungrammatical. In the present study, we compared the ERP responses associated with an ungrammatical gendered demonstrative pronoun (*éste)* and a correct gendered pronoun (*ésta)*. The results showed an ungrammatical morphosyntactic mismatch for *éste* in the form of a LAN response. Hence, in our opinion, the observed frontal negativity and LAN responses reflect different cognitive processes. In short, a LAN is observed in response to referential difficulties only when the sentence is ungrammatical (i.e., *éste vs. ésta*), but LAN was observed for morphosyntactic gender disagreements (i.e., *esto vs. ésta*). Therefore, we believe that our observed frontal negativity to *esto* reflects the cost of creating a new reference from the reactivation of information in the working memory (different from the late positivity that reflects a cognitive costs to an update in the mental model). Namely, in our case, *esto* does not refer to multiple possible antecedents. Instead, *esto* refers to a context with a greater amount of information that must be determined to identify and link with the discourse. In accordance with our interpretation, Almor and Eimas (2008) showed a frontal negativity when activating memory representations from previous information of the discourse.

### P200

The neuter demonstrative pronoun *esto*, and the gendered demonstrative pronoun *ésta*, yielded an early positivity. Specifically, *ésta* elicited a larger positive deflection around 200 ms (i.e., P200) when compared with *esto*. Relatively little is known about the P200’s relationship with language-processing, and has been implicated in a variety of contexts. Namely, a larger P200 has been observed with respect to semantic expectancy in linguistic contexts or sentence processing (Federmeier and Kutas, 2002; Federmeier et al., 2005; Ferretti et al., 2007), word repetition in discourse (van Petten et al., 1991), and pragmatic effects associated with verb factivity in discourse processing (Ferretti et al., 2008). Increased P200 amplitudes have been thought to reflect enhanced retrieval (Smith, 1993; Dunn et al., 1998), or the encounter of unexpected or improbable stimuli (Peters et al., 2005).

Further, van Berkum et al. (2005) presented written stories intended to lead the participants to predict a specific gendered noun. Adjectives introduced into the story either matched, or mismatched, the gender of the predicted noun. Accordingly, mismatching adjectives elicited a larger P200 than matching adjectives. These data suggest that the P200 may be sensitive to graphic form of written language. In other words, the presented nouns created a visual expectation of the grapheme needed to mark the adjective’s gender, leading the matching adjectives to be more expected, and thus explaining the smaller P200. In support of this idea, Hung and Schumacher (2012) saw a reduced P200 for co-referential Chinese characters when preceded by a graphically similar character. The authors argue that repetition of a previously encountered graphic form permits a quick retrieval and decoding of incoming input (i.e., smaller P200), while introducing new graphic forms creates a perceptual obstacle in co-reference (i.e., larger P200) (Liu et al., 2003; Hung and Schumacher, 2014).

However, graphical similarity between a demonstrative pronoun and its antecedent cannot explain our observed P200 amplitude trends in the same way. Explicitly, we observed a larger P200 to anaphoric relationships in which the antecedent shared more graphical features, and thus could clearly predict the pronoun’s gender (feminine object- *renuncia*; feminine pronoun- *ésta*) compared to when there was no clear predictive relationship (i.e., event- *renuncia fue aceptada*; neuter pronoun- *esto*). Fortunately, there is another point of view that accounts for how top-down constraints on the visual processing of upcoming stimuli modulate the P200 amplitude (Federmeier and Kutas, 2002; Federmeier et al., 2005; Ferretti et al., 2007, 2008; Yang et al., 2019). Specifically, since words can be identified within the first 200 ms during sentence reading (Dambacher et al., 2006), the P200 amplitude can increase for more predictable words relative to words that are less likely (Federmeier et al., 2005). Thus, given the strong predictive anaphoric relationship between a gendered antecedent and a pronoun of the same gender, we could expect to see a larger P200 for *ésta*, compared to *esto*. Our findings extend this idea to anaphoric resolution, such that the relative ability of an antecedent to predict a given demonstrative pronoun modulates the P200.

**FIGURE 3:**
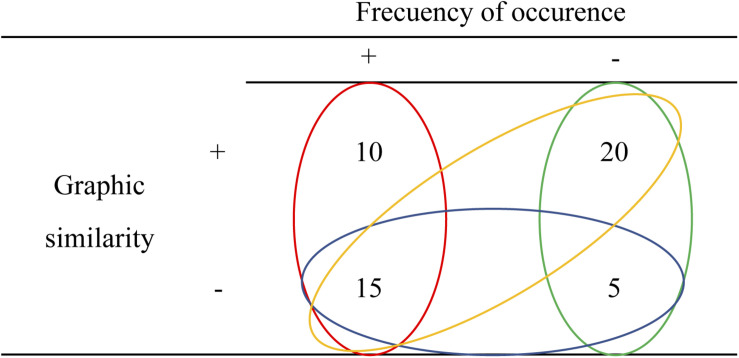
A cross table of all possible results in the frequency of occurrence and graphic similarity interaction. Numbers represent the trends reported in previous research concerning the P200 amplitude modulation (see text for references) and circles represent the comparisons previously reported. Yellow circle represents comparison *ésta* (20) vs. *esto* (15). Green circle: *ésta* (20) vs. *éste* (5). Red circle: identity relation (10) – same word context and anaphora– vs. *ésto* (15). Blue circle: *esto* (15) vs. *éste* (5). pronoun resolution, P200, N400.

Predictability of the demonstrative pronouns can be assessed by a Cloze task to support its association with amplitude’s modulation of P200. Unfortunately, Cloze task was not collected in the present study, thus being a limitation in our study. However, the frequency of occurrence of the pronouns tested in the present investigation can be obtained from the Spanish lexical database base (see text footnote 1). The lexical database showed that the frequency of occurrence of *esto* (728 per million) is higher than that of *ésta* (169.66 per million), which makes our interpretation somewhat contradictory. However, if we combine the previous findings and ours, and consider that the P200 is modulated as a result of the interaction between frequency of occurrence and graphic similarity, our results are no longer contradictory. Using Figure 3 (an interaction table of all possible results) as a guide, it can be seen that if word frequency is the only variable manipulated, then a larger P200 is observed for the more expected word (Federmeier and Kutas, 2002; Federmeier et al., 2005; Ferretti et al., 2007, 2008; Yang et al., 2019; Figure 3 blue circle). On the other hand, if graphic similarity is the only variable manipulated, then a smaller P200 amplitude is observed when the antecedent and the anaphor are the same word than when they are different words (van Berkum et al., 2005; Hung and Schumacher, 2012, Hung and Schumacher, 2014; Burmester et al., 2014; Figure 3 red circle).

With this in mind, an interaction between graphic similarity and frequency of occurrence can explain the results in the present study. Again, using Figure 3 as a guide, it can be seen that our results go in the expected direction and add relevant information in the P200 literature. Precisely, in the case that the anaphora has a demonstrative pronoun that matches the gender of the antecedent (higher graphic similarity) but is less expected (less frequency of occurrence), a larger P200 is observed compared to when a demonstrative mismatches the gender (less graphic similarity) but is more predictable (value 20 vs. value 15 in Figure 3 yellow circle). This respectively, corresponds to our *ésta* (low frequency and high graphic similarity) vs. *esto* (high frequency and low similarity) comparison and results (larger P200 for *ésta*).

Furthermore, an anaphora with graphical similarity but less frequent demonstrative as described above, elicits a larger P200 compared to an anaphora with a demonstrative that mismatches the gender of the antecedent (less graphic similarity) and with a null predictability (value 20 vs. value 5 in Figure 3 green circle). This respectively, corresponds to our *ésta* (low frequency and high graphic similarity) vs. *éste* (low frequency and low similarity) comparison and results (larger P200 for *ésta*). Thus, these findings suggest that the anaphora is graphically processed (morphosyntactic characteristics) before 200 ms and the referential relationship is provided based on lexical expectations formed by the context.

Without further research, we can only speculate on the reason for these effects on the P200. According to the Bayesian framework (Kehler and Rohde, 2019), comprehenders form predictions about which referent is likely to be mentioned again later in the discourse, based on the content of the prior discourse. When comprehenders find an anaphora, they update their prediction of which is the referent by integrating their initial predictions (prior) with the referential bias (evidence) that is given by the form of the anaphora. We believe that the P200 effect may reflect an early update of the prediction involving graphical features.

## Conclusion

By examining ERP responses to two types of anaphoric relationships between demonstrative pronouns and their antecedents, we have provided evidence that before 200 ms (after onset of the anaphora presentation) morphosyntactic features are processed based on the lexical expectations formed by reading the context (P200 effect). These expectations can initiate a rapid mapping of the pronoun’s gender suffixes to possible antecedents. This process is followed by a frontal negativity (rather than the classic parietal N400), which represents referential resolution processing efforts in expressions that require retrieving a complete clause memory and linking with a neutral demonstrative. In contrast, the LAN effect represents the cognitive cost of linking an anaphor and its antecedent that disagree in morphosyntactic gender and create a grammatical violation.

## Data Availability Statement

Data are available at: https://doi.org/10.6084/m9.figshare.14161568.

## Ethics Statement

The studies involving human participants were reviewed and approved by the Ethics Committee of Universidad Nacional Autónoma de México (Ethical Application Ref: CE/FESI/062020/1299). The patients/participants provided their written informed consent to participate in this study.

## Author Contributions

AG-S and JS-P conceived and designed the study. GA-C oversaw the implementation of the study and data collection. AG-S carried out the statistical analyses. AG-S, NW, GA-C, and JS-P wrote the manuscript. All authors contributed to the article and approved the submitted version.

## Conflict of Interest

The authors declare that the research was conducted in the absence of any commercial or financial relationships that could be construed as a potential conflict of interest.

## Footnotes

1 https://www.bcbl.eu/databases/espal/
