# Supplementary material for: An Event-Related Brain Potential (ERP) Study of Complex Anaphora in Spanish
Source: Front Psychol. 2021 Mar 18;12:625314. doi: 10.3389/fpsyg.2021.625314 (PMC8012726; doi:10.3389/fpsyg.2021.625314)
Supplement: Supplementary file 1 [file Table_1.pdf]

APENDIX A

Target sentences

A list contained 40 sentences with ésta, esto, and éste (120 unique sentences)

1	La	renuncia	fue	efectiva	pero	ésta/esto/éste	no	sorprendió	al		empresario
2	La	actuación	estuvo	maravillosa	pero	ésta/esto/éste	no	impactó	al		público
3	La	película	estuvo	fascinante	pero	ésta/esto/éste	no	impresionó	a	mi	hermano
4	La	botella	estuvo	estrellada	pero	ésta/esto/éste	no	preocupó	al		mesero
5	La	pistola	estuvo	cargada	pero	ésta/esto/éste	no	amedrentó	al		jugador
6	La	nave	estuvo	rodeada	pero	ésta/esto/éste	no	contuvo	al		capitán
7	La	casa	estuvo	cerrada	pero	ésta/esto/éste	no	impidió		el	robo
8	La	ballesta	estuvo	descompuesta	pero	ésta/esto/éste	no	descalificó	al		equipo
9	La	boina	estuvo	perdida	pero	ésta/esto/éste	no	incomodó	al		militar
10	La	ampliadora	estuvo	descompuesta	pero	ésta/esto/éste	no	retrasó	al		fotógrafo
11	La	computadora	estuvo	lentificada	pero	ésta/esto/éste	no	suspendió		la	tarea
12	La	colcha	estuvo	sucia	pero	ésta/esto/éste	no	levantó	a	la	dama
13	La	navaja	estuvo	oxidada	pero	ésta/esto/éste	no	asombró	al		experto
14	La	barriga	estuvo	inflada	pero	ésta/esto/éste	no	molestó	a	mi	novia
15	La	axila	estuvo	olorosa	pero	ésta/esto/éste	no	ahuyentó	a	la	muchacha
16	La	puerta	estuvo	cerrada	pero	ésta/esto/éste	no	disminuyó		el	ruido
17	La	uña	estuvo	enterrada	pero	ésta/esto/éste	no	dolió	al		atleta
18	La	bota	fue	entintada	pero	ésta/esto/éste	no	satisfizo	al		cliente
19	La	planta	estuvo	seca	pero	ésta/esto/éste	no	enojó	a	la	señora
20	La	moneda	estuvo	deslumbrante	pero	ésta/esto/éste	no	ocultó		su	falsedad
21	La	enfermedad	estuvo	erradicada	pero	ésta/esto/éste	no	sirvió	gran		cosa
22	La	medalla	fue	resaltada	pero	ésta/esto/éste	no	ayudó	al		ganador
23	La	tumba	fue	pisoteada	pero	ésta/esto/éste	no	manchó		el	honor
24	La	espada	estuvo	arrumbada	pero	ésta/esto/éste	no	disminuyó		su	filo
25	La	silla	estuvo	maltratada	pero	ésta/esto/éste	no	impidió		su	uso
26	La	pila	fue	recargada	pero	ésta/esto/éste	no	resolvió		el	problema
27	La	roca	fue	destruida	pero	ésta/esto/éste	no	evitó		la	avalancha
28	La	locomotora	estuvo	retrasada	pero	ésta/esto/éste	no	alteró		su	llegada
29	La	margarita	estuvo	marchita	pero	ésta/esto/éste	no	disminuyó		su	belleza
30	La	motocicleta	fue	engrasada	pero	ésta/esto/éste	no	mejoró		su	velocidad
31	La	sala	fue	renovada	pero	ésta/esto/éste	no	embelleció		la	casa

31	La	falda	estuvo	manchada	pero	ésta/esto/éste	no	apenó	a	la	muchacha
33	La	butaca	estuvo	embarrada	pero	ésta/esto/éste	no	ensució	al		caballero
34	La	frase	estuvo	incompleta	pero	ésta/esto/éste	no	importó	al		gobernador
35	La	carpeta	estuvo	etiquetada	pero	ésta/esto/éste	no	ayudó	al		secretario
36	La	avioneta	fue	abordada	pero	ésta/esto/éste	no	aumentó		la	seguridad
37	La	huerta	estuvo	abandonada	pero	ésta/esto/éste	no	perjudicó		la	cosecha
38	La	libreta	fue	pintada	pero	ésta/esto/éste	no	enojó	a	la	maestra
39	La	jarra	estuvo	llena	pero	ésta/esto/éste	no	sació		su	sed
40	La	lata	estuvo	perforada	pero	ésta/esto/éste	no	limitó		su	uso
41	La	grieta	estuvo	arreglada	pero	ésta/esto/éste	no	provocó		el	hundimiento
42	La	lancha	fue	averiada	pero	ésta/esto/éste	no	detuvo	al		policía
43	La	catedral	estuvo	cerrada	pero	ésta/esto/éste	no	complicó	al		turista
44	La	taza	estuvo	vacía	pero	ésta/esto/éste	no	disgustó	al		alcohólico
45	La	cruz	fue	apuntalada	pero	ésta/esto/éste	no	retuvo		su	caída
46	La	herramienta	fue	contaminada	pero	ésta/esto/éste	no	perjudicó	al		paciente
47	La	trompeta	estuvo	brillante	pero	ésta/esto/éste	no	llamó		la	atención
48	La	arcilla	estuvo	amasada	pero	ésta/esto/éste	no	sirvió	al		escultor
49	La	feria	estuvo	concurrida	pero	ésta/esto/éste	no	aumentó		las	ventas
50	La	estrella	fue	observada	pero	ésta/esto/éste	no	contribuyó	al		conocimiento
51	La	radio	estuvo	prendida	pero	ésta/esto/éste	no	incrementó		la	audiencia
52	La	azotea	estuvo	mojada	pero	ésta/esto/éste	no	trasminó		el	techo
53	La	banqueta	estuvo	ocupada	pero	ésta/esto/éste	no	impidió		el	tránsito
54	La	sortija	estuvo	hechizada	pero	ésta/esto/éste	no	asustó	a	la	princesa
55	La	cerradura	estuvo	asegurada	pero	ésta/esto/éste	no	detuvo	al		ladrón
56	La	cuchara	estuvo	sucia	pero	ésta/esto/éste	no	obstaculizó		el	almuerzo
57	La	pipa	estuvo	encendida	pero	ésta/esto/éste	no	tranquilizó	al		chamán
58	La	mochila	estuvo	abierta	pero	ésta/esto/éste	no	incitó		el	robo
59	La	bolsa	fue	rasgada	pero	ésta/esto/éste	no	tiró		su	contenido
60	La	piel	estuvo	seca	pero	ésta/esto/éste	no	estimuló		el	ardor
61	La	cerveza	estuvo	caliente	pero	ésta/esto/éste	no	limitó	al		bebedor
62	La	maceta	estuvo	rota	pero	ésta/esto/éste	no	dejó	escapar	el	agua
63	La	jugada	fue	descubierta	pero	ésta/esto/éste	no	dio		el	triunfo
64	La	novela	estuvo	aburrida	pero	ésta/esto/éste	no	impidió		su	lectura
65	La	melena	fue	tratada	pero	ésta/esto/éste	no	sostuvo		su	caída
66	La	astilla	estuvo	enterrada	pero	ésta/esto/éste	no	molestó	a	mi	hijo

67	La	sal	estuvo	húmeda	pero	ésta/esto/éste	no	dificultó	su		salida
68	La	cena	estuvo	servida	pero	ésta/esto/éste	no	alteró	su		sabor
69	La	guitarra	estuvo	desafinada	pero	ésta/esto/éste	no	repercutió	en	el	concierto
70	La	naranja	fue	partida	pero	ésta/esto/éste	no	lastimó	a		nadie
71	La	pulsera	estuvo	empeñada	pero	ésta/esto/éste	no	acumuló		mucho	interés
72	La	charla	fue	malinterpretada	pero	ésta/esto/éste	no	afectó		la	relación
73	La	caricatura	fue	utilizada	pero	ésta/esto/éste	no	ridiculizó		a	nadie
74	La	bocina	estuvo	apagada	pero	ésta/esto/éste	no	silenció		la	tocada
75	La	habitación	estuvo	decorada	pero	ésta/esto/éste	no	resultó			apropiado
76	La	cuerda	fue	cortada	pero	ésta/esto/éste	no	provocó		el	accidente
77	Mi	pierna	estuvo	lesionada	pero	ésta/esto/éste	no	imposibilitó		que	corriera
78	La	medicina	fue	empleada	pero	ésta/esto/éste	no	motivó			mejoría
79	La	pintura	estuvo	embarrada	pero	ésta/esto/éste	no	necesitó		de	solvente
80	La	pelota	estuvo	desinflada	pero	ésta/esto/éste	no	explicó		tu	enojo
81	La	plata	estuvo	deslumbrante	pero	ésta/esto/éste	no	disimuló		las	abolladuras
82	La	pared	estuvo	agujerada	pero	ésta/esto/éste	no	pareció	después	de	pintarla
83	La	cama	estuvo	destendida	pero	ésta/esto/éste	no	indujo	a		usarla
84	La	vasija	estuvo	descarapelada	pero	ésta/esto/éste	no	dificultó		su	venta
85	La	flor	estuvo	seca	pero	ésta/esto/éste	no	afectó		su	olor
86	La	chaqueta	estuvo	rota	pero	ésta/esto/éste	no	obligó		a	coserla
87	La	alfombra	estuvo	polvorienta	pero	ésta/esto/éste	no	ensució		tu	pantalón
88	La	almendra	estuvo	amarga	pero	ésta/esto/éste	no	arruinó		el	gisado
89	La	familia	estuvo	reunida	pero	ésta/esto/éste	no	justificó		el	festejo
90	La	montaña	estuvo	nevada	pero	ésta/esto/éste	no	obstaculizó		el	ascenso
91	La	maleta	estuvo	cerrada	pero	ésta/esto/éste	no	evitó	que	faltara	dinero
92	La	diligencia	fue	rápida	pero	ésta/esto/éste	no	eludió	que	la	asaltaran
93	La	luna	estuvo	deslumbrante	pero	ésta/esto/éste	no	asustó	a	la	población
94	La	comida	estuvo	deliciosa	pero	ésta/esto/éste	no	extinguió		el	apetito
95	La	mandolina	estuvo	afinada	pero	ésta/esto/éste	no	mostró	su	buen	tono
96	La	cuenca	estuvo	inundada	pero	ésta/esto/éste	no	lesionó	a	los	habitantes
97	La	conferencia	estuvo	concurrida	pero	ésta/esto/éste	no	complació	al		exponente
98	La	vereda	estuvo	cerrada	pero	ésta/esto/éste	no	arriesgó	al		viajero
99	La	ropa	estuvo	tendida	pero	ésta/esto/éste	no	decoloró		su	tejido
100	La	ventana	estuvo	abierta	pero	ésta/esto/éste	no	empolvó		el	salón
101	La	cómoda	estuvo	asoleada	pero	ésta/esto/éste	no	maltrató		su	barniz

102	La	aguja	estuvo	ensartada	pero	ésta/esto/éste	no	sirvió	para	el	bordado
103	La	fotografía	estuvo	perdida	pero	ésta/esto/éste	no	duró		mucho	tiempo
104	La	disculpa	estuvo	exagerada	pero	ésta/esto/éste	no	ameritó		el	perdón
105	La	muñeca	estuvo	desgreñada	pero	ésta/esto/éste	no	decepcionó	a	la	niña
106	La	charca	estuvo	contaminada	pero	ésta/esto/éste	no	causó		una	epidemia
107	La	pantalla	estuvo	instalada	pero	ésta/esto/éste	no	posibilitó		la	visualización
108	La	perla	fue	encontrada	pero	ésta/esto/éste	no	ocurrió	en	poco	tiempo
109	La	derrota	estuvo	anunciada	pero	ésta/esto/éste	no	evitó		que	sucediera
110	La	camioneta	estuvo	guardada	pero	ésta/esto/éste	no	descargó		el	acumulador
111	La	cadera	estuvo	rota	pero	ésta/esto/éste	no	lastimó		al	fémur
112	La	arena	estuvo	amontonada	pero	ésta/esto/éste	no	ameritó	sancionar	al	ingeniero
113	La	gráfica	estuvo	equivocada	pero	ésta/esto/éste	no	provocó	usar	el	corrector
114	La	revista	estuvo	vetada	pero	ésta/esto/éste	no	obstaculizó		su	circulación
115	La	fragata	estuvo	anclada	pero	ésta/esto/éste	no	desalentó		al	viajero
116	La	pradera	estuvo	sembrada	pero	ésta/esto/éste	no	sirvió	al		campesino
117	La	ginebra	fue	desperdiciada	pero	ésta/esto/éste	no	terminó		la	reunión
118	La	calle	estuvo	bloqueada	pero	ésta/esto/éste	no	provocó		el	tráfico
119	La	fuga	estuvo	planeada	pero	ésta/esto/éste	no	llevó	al		éxito
120	La	máquina	estuvo	parada	pero	ésta/esto/éste	no	detuvo		el	pedido

Filler sentences

A list contained 180 unique filler sentences

1	La	sociedad	de	astronomí a	organizó		una	reunión	Propuso	nuevos		principios
2	La	compañía		eléctrica	reparó	la	estación	central	Arreglaron	un	viejo	transformador
3	Los	papás			cenaron	con	sus	amigos	Rió	toda	la	noche
4	El	marino			apresó	al		enemigo	Atacaron		por	sorpresa
5	El	crimen			planeó	su		ataque	Asaltó	un	centro	comercial
6	El	achichincle			alabó	al		jefe	Exageró		su	actuación
7	La	orquesta			tocó	por	la	noche	Seleccionó	varias	piezas	clásicas
8	El	senado			superó		la	controversia	Votaron	una	nueva	ley
9	Las	aeromozas			ofrecieron			champaña	Sirvió		en	abundancia
10	Los	requintos			interpretaron		la	melodía	Cantaron		con	armonía
11	La	IBM			generó	un	buen	algoritmo	Descifraron	un	código	encriptado
12	Los	universitarios			recibieron		un	premio	Impusieron		nuevo	record
13	El	elefante			tiró	los		árboles	Huyó	del		fuego
14	El	pez			evitó	a	la	foca	Nadaron		muy	veloz
15	Los	cantantes			interpretaron		la	canción	Acopló		sus	tonos
16	El	coro			interpretó		una	ópera	Cantó	en	el	teatro
17	El	árbol			desvió		el	rio	Protegió		la	casa
18	El	pasajero			apretó	al		niño	Ocasionaron		el	accidente
19	Los	egresados	de	secundaria	organizaron		la	fiesta	Coordinaron		la	asistencia
20	El	proletariado			denunció		la	injusticia	Inició		una	huelga
21	La	isla			apareció	en	el	radar	Asustó		al	navegante
22	Los	milicianos			avanzaron	por	el	bosque	Escondieron		el	armamento
23	La	jauría			alcanzó	a	la	expedición	Mordió	al		guía
24	La	familia			descubrió		la	travesura	Castigó	al		niño
25	La	uva			Aportó	un	delicado	sabor	Convencieron	al		experto
26	Los	ayudantes			apoyaron		al	delegado	Demostró		su	honestidad
27	El	partido		comunista	redactó		el	manifiesto	Actualizaron	su	visión	política
28	El	alumno			exigió	sus		derechos	Presionó	al		director
29	La	tropa			corrió	entre	los	árboles	Prepararon	una		emboscada
30	El	jinete			alcanzó		su	destino	Atravesaron	el	caluroso	desierto
31	El	ejército			guardó		el	orden	Protegió	a	la	población
32	El	opus	Dei		revivió	la	pasión	católica	Rezaron	durante	la	semana
33	Los	comerciales			anunciaron	un	nuevo	plan	Bajaron	todos	los	precios
34	La	abeja			persiguió	al		jardinero	Picó	en	la	espalda
35	La	tripulación			completó		la	misión	Llegó	a	la	luna
36	La	congregación			promovió		una	colecta	Consiguieron	mucha		ropa
37	El	obrero			emplazó		la	huelga	Presentaron	su	pliego	petitorio

38	El	comando			mató	al		disidente	Gastó	dinero	del	dictador
39	La	maestra			expulsó	al	niño	travieso	Llamó	a	los	padres
40	La	hormiga			invadió		la	cocina	Subió		al	pastel
41		Soriana			clasificó	la	nueva	mercancía	Comprobaron		los	precios
42	La	comunidad		Científica	estudió		el	problema	Rechazó		la	teoría
43	El	alumnado	de	teatro	envolvió	al		espectador	Divirtieron		al	público
44	El	individuo			corrió	por	la	calle	Interrumpieron		el	tránsito
45	La	semilla			generó		una	planta	Mostraron		su	efectividad
46	La	SEAT			ofreció		una	promoción	Añadieron	el	aire	acondicionado
47	Los	secretarios	de	estado	sesionaron	con	el	presidente	Ahondaron		el	divisionismo
48	La	clase		política	visitó		una	escuela	Alentó	a	los	niños
49	El	profesor			modificó		el	temario	Completaron		el	contenido
50	Los	monjes			cosecharon		la	uva	Produjeron		el	vino
51	El	policía			investigó		el	homicidio	Revisó		el	expediente
52	El	ayuntamiento			atendió	a	las	exigencias	Regularon		las	tasas
53	La	bandada			voló	en	la	noche	Emigró	hacia	el	norte
54	El	pájaro			viajó	al		norte	Voló		al	atardecer
55	El	congreso			concluyó	con	una	propuesta	Trazaron	una	nueva	línea
56	El	estudiante			acató		la	disposición	Retiró		su	demanda
57	El	funcionario		fiscal	ocultó		la	información	Recaudó	más		dinero
58	Los	funcionarios			auditaron		al	municipio	Revisó		sus	gastos
59	El	tenista			participó	en	la	contienda	Ganaron	todos	los	juegos
60	El	jurado	realizó	las	sesiones	en	el	juzgado	Declaró	culpable	al	preso
61	La	orden		franciscana	renunció		a	hablar	Hicieron	voto	de	silencio
62	Los	feligreses			visitaron		los	hogares	Invitó	a	la	convivencia
63	Los	delegados			hicieron		el	calendario	Incorporó		nuevos	equipos
64	Las	religiosas			rezaron	toda	la	noche	Pidió	por	la	paz
65	El	Sindicato			protestó	por	el	salario	Manifestó	sus		demandas
66	La	audiencia			excluyó	la	evidencia	principal	Inculparon	al	feroz	asesino
67	Los	salvajes			grafitearon		mi	casa	Pintaron			obscenidades
68	El	municipio			sostuvo		su	reforma	Facilitaron	el	trámite	burocrático
69	El	defensa			paró		el	ataque	Desvió	el		tiro
70	La	CIA			ubicó	al		terrorista	Interrogaron	duramente	al	prisionero
71	Los	empresarios			cambiaron	su		estrategia	Invirtieron	en	la	agricultura
72	El	soldado			saludó	al		comandante	Agachó		la	cabeza
73	La	gente			habla	mucho	de	fútbol	Mira	todos	los	partidos
74	El	delantero			brindó	un	buen	espectáculo	Goleó	al	otro	equipo
75	La	oveja			saltó		la	cerca	Corrió		al	monte
76	Los	guitarristas			amenizaron		la	tarde	Logró	un	gran	éxito
77	La	tripulación			atracó			anoche	Navegó	hasta	la	isla
78	El	gobierno			debatió		la	reforma	Aceptó	pero	con	cambios
79	El	bato			llegó	al		antro	Hicieron		gran	estruendo

80	La	Gerencia			autorizó	un	pago	adelantado	Absorbieron		los	impuestos
81	Las	naciones			alcanzaron		un	pacto	Negoció		sus	propuestas
82	El	directivo			canceló	las	cuentas	ilícitas	Obedeció	al		Ministerio
83	El	carabinero			recibió	la		señal	Disparó	al		traidor
84	La	monarquía			firmó	un	real	decreto	Confirmaron	la	propuesta	gubernamental
85	Los	inversionistas			vendieron		sus	acciones	Recuperó		sus	pérdidas
86	La	Honda			alcanzó	el	mejor	tiempo	Conquistaron	el		campeonato
87	El	energúmeno			entró	a	la	alberca	Alteró		el	orden
88	Los	actores			salieron	a		presentarse	Recibió		muchos	aplausos
89	El	consejo			insistió	en	la	prevención	Recomendaron	ahorrar		agua
90	Los	paramilitares			capturaron		al	rebelde	Consiguió		su	rendición
91	El	partido		dominante	reclamó	a	la	oposición	Averiguaron	algunas		irregularidades
92	El	piloto			hizo	la	mejor	vuelta	Venció	al		oponente
93	Los	capitanes			dejaron		la	base	Abandonó		su	posición
94	El	partido		político	apoyó		la	elección	Promovió	el		voto
95	El	espectador			aplaudió	al		tenor	Apreció		su	interpretación
96	El	explorador			alcanzó		la	cima	Vieron	realizado	su	sueño
97	Los	cristianos			convencieron	a	los	incrédulos	Engrandeció		los	milagros
98	Los	campesinos			sembraron	el		trigo	Descansaron	en	el	granero
99	Las	chelistas			comenzaron		su	interpretación	Tuvo		un	desentono
100	El	sirviente			limpió		la	mansión	Prepararon		el	festejo
101	Los	aliados			hicieron		el	manifiesto	Declararon		sus	principios
102	El	asistente			elogió	al		expositor	Aplaudieron		con	entusiasmo
103	Los	investigadores			descubrieron		el	fósil	Revisaron		el	acantilado
104	La	marina			encontró		un	náufrago	Participó	en	el	rescate
105	El	tribunal			prohibió		la	enmienda	Protegieron	los	intereses	populares
106	El	narco			introdujo		la	cocaína	Burlaron		la	vigilancia
107	Unas	mujeres	del	sultán	bailaron		la	danza	Estimularon	a	los	asistentes
108	Los	académicos			revisaron		el	protocolo	Encontraron		su	viabilidad
109	El	jugador			apretó	al		final	Ganó		la	partida
110	El	equipo		médico	operó	a	corazón	abierto	Empleó	una	nueva	técnica
111	El	agente			sospechó		del	conductor	Midió		la	velocidad
112	La	colmena			vivía	en	un	peral	Produjo	mucha		miel
113	El	fiel			indujo		el	sacrificio	Mataron	al		gallo
114	El	ganado			comió	en	el	pastizal	Caminó	entre	las	colinas
115	La	avispa			anidó	en	el	techo	Tapó		la	ventilación
116	La	persona			declaró		su	malestar	Gritaron		sus	consignas
117	Los	familiares			llegaron	al		cementerio	Mostró		su	dolor
118	Los	opositores			bloquearon		la	sesión	Tomaron		la	tribuna
119	La	guerrilla			entró	en	la	selva	Luchó	contra	el	ejército
120	El	activista			organizó	un	acto	político	Filmaron		su	mitin
121	El	estudiante			reconoció	al		catedrático	Citó	su		trabajo

122	El	cerdo			salió	del		chiquero	Olió		su	alimento
123	La	compañía	de	danza	caracterizó	una	obra	moderna	Bailaron	con	movimientos	rápidos
124	Los	abogados			objetaron		la	sentencia	Encontró		unas	incongruencias
125	La	confederación		mercantil	cambió	su		estrategia	Invirtió	en	la	agricultura
126	La	tecla			desconfiguró		la	computadora	Bloquearon		el	proceso
127	Los	proletarios			redujeron		su	ingreso	Participaron	en	el	movimiento
128	Las	bastoneras			impresionaron	a	la	multitud	Ejecutó	bien	sus	acrobacias
129	Unos	guitarristas	de	jazz	estuvieron	en		Málaga	Tocaron	durante	una	hora
130	El	batallón			penetró		las	trincheras	Desplazó	al		enemigo
131	El	barco			rodeó	la		península	Navegó	toda	la	noche
132	Las	monjas			hicieron		el	rompope	Utilizó	los	mejores	ingredientes
133	Los	educadores			agruparon	a	los	niños	Investigó		sus	capacidades
134	El	solista			interpretó		la	obra	Entonaron	con	gran	maestría
135	El	rufián			escribió		sus	memorias	Redactaron	todas	sus	canalladas
136	La	comisión	nacional	bancaria	pactó	con	la	administración	Compensaron		al	pueblo
137	La	hoja			sirvió	a	las	hormigas	Proporcionó		su	alimento
138	Los	diputados			propusieron		una	ley	Buscó		el	consenso
139	Los	agremiados			siguieron		el	estatuto	Convocaron	a	una	junta
140	Los	ingenieros			modificaron		el	túnel	Aumentó		su	presupuesto
141	Los	defensas			jugaron	en	el	estadio	Ganaron		el	partido
142	La	expedición			nadó	hacia	el	coral	Huyeron	del		tiburón
143	Los	tesoreros			finalizaron	toda	su	gestión	Presentaron		las	finanzas
144	El	equipo	de	rescate	subió	a	la	montaña	Salvó	al		niño
145	El	fiel			llegó	a	su	destino	Caminó		sin	fatiga
146	El	creyente			celebró	un	acto	religioso	Oraron		con	devoción
147	Los	banqueros			hablaron	con	el	gobierno	Financiaron	un	proyecto	social
148	El	agremiado			protestó	contra	el	empresario	Propusieron		la	huelga
149	Los	amotinados			tomaron		varios	rehenes	Despistó	a	la	vigilancia
150	Los	fisiólogos			modificaron		el	temario	Encontró		muchas	repeticiones
151	El	clero			juzgó		la	blasfemia	Excomulgó	a	un	filósofo
152	Los	burócratas			recibieron		la	sanción	Rebasó		la	norma
153	Los	hermanos			defendieron	al		muchacho	Explicaron		sus	razones
154	El	vecino			organizó		una	posada	Adornaron		las	calles
155	Los	químicos			recibieron		sus	reactivos	Terminó		su	investigación
156	El	circo			provocó		el	asombro	Lograron		algo	increíble
157	Los	científicos			apoyaron		la	teoría	Comprobó		sus	hipótesis
158	Los	jóvenes			valoraron	a	sus	padres	Comprendieron		su	esfuerzo
159	El	cliente			aprovechó	las		rebajas	Compró	la		verdura
160	El	sardo			recibió		las	instrucciones	Desfilaron		con	marcialidad
161	El	compinche	del	presidente	utilizó		el	presupuesto	Derrocharon	en	sus	pachangas

162	La	burguesía			acumuló	su		riqueza	Explotaron	al		obrero
163	La	compañía	de	teatro	adaptó		la	obra	Representaron		un	drama
164	Los	asesores			invirtieron		en	metales	Aseguró		las	ganancias
165	El	profesorado			caricaturizó	a	los	diputados	Hablaron	sobre	la	honradez
166	Los	clubes	de	futbol	atrajeron	a		patrocinadores	Incrementaron		sus	ganancias
167	El	edificio			desaguó		al	río	Contaminaron		el	ambiente
168	Los	alumnos	de	secundaria	tuvieron	mal		comportamiento	Hablaron	todo	la	mañana
169	Los	mecánicos			arreglaron	mi		coche	Trabajaron	todo	el	día
170	Los	camaradas			iniciaron	al		nuevo	Impusieron		su	reglamento
171	El	soldado			invadió		el	país	Tomaron		sus	ciudades
172	Los	guardianes	del	Rey	atraparon	al		conspirador	Descubrieron		sus	intenciones
173	Los	curas		católicos	mantuvieron	su		posición	Predicaron	contra	el	aborto
174	La	editorial			trabajó	en	el	libro	Publicó	una	nueva	edición
175	Los	industriales			incrementaron		la	inversión	Abrió		más	talleres
176	El	descendiente			defendió		al	padre	Explicaron		su	conducta
177	La	manada			corrió	en		estampida	Destruyó	una		villa
178	La	universidad			procuró		la	convergencia	Proporcionaron		información	adecuada
179	El	escritor			inauguró		el	congreso	Dictaron	una	conferencia	magistral
180	Los	esposos			escogieron		los	anillos	Recorrieron		varias	joyerías
